# A new experimental setup to measure hydraulic conductivity of plant segments

**DOI:** 10.1093/aobpla/plad024

**Published:** 2023-06-15

**Authors:** Louis Krieger, Stanislaus J Schymanski

**Affiliations:** Environmental Research and Innovation, Luxembourg Institute of Science and Technology, 41 Rue du Brill, 4422 Belvaux, Luxembourg; Faculty of Science, Technology and Medicine, University of Luxembourg, 2 Av. de l’Universite, 4365 Esch-sur-Alzette, Luxembourg; Environmental Research and Innovation, Luxembourg Institute of Science and Technology, 41 Rue du Brill, 4422 Belvaux, Luxembourg; Faculty of Science, Technology and Medicine, University of Luxembourg, 2 Av. de l’Universite, 4365 Esch-sur-Alzette, Luxembourg

## Abstract

Plant hydraulic conductivity and its decline under water stress are the focal point of current plant hydraulic research. The common methods of measuring hydraulic conductivity control a pressure gradient to push water through plant samples, submitting them to conditions far away from those that are experienced in nature where flow is suction driven and determined by the leaf water demand. In this paper, we present two methods for measuring hydraulic conductivity under closer to natural conditions, an artificial plant setup and a horizontal syringe pump setup. Both approaches use suction to pull water through a plant sample while dynamically monitoring the flow rate and pressure gradients. The syringe setup presented here allows for controlling and rapidly changing flow and pressure conditions, enabling experimental assessment of rapid plant hydraulic responses to water stress. The setup also allows quantification of dynamic changes in water storage of plant samples. Our tests demonstrate that the syringe pump setup can reproduce hydraulic conductivity values measured using the current standard method based on pushing water under above-atmospheric pressure. Surprisingly, using both the traditional and our new syringe pump setup, we found a positive correlation between changes in flow rate and hydraulic conductivity. Moreover, when flow or pressure conditions were changed rapidly, we found substantial contributions to flow by dynamic and largely reversible changes in the water storage of plant samples. Although the measurements can be performed under sub-atmospheric pressures, it is not possible to subject the samples to negative pressures due to the presence of gas bubbles near the valves and pressure sensors. Regardless, this setup allows for unprecedented insights into the interplay between pressure, flow rate, hydraulic conductivity and water storage in plant segments. This work was performed using an Open Science approach with the original data and analysis to be found at https://doi.org/10.5281/zenodo.7322605.

## Background and Aims

Plant leaves absorb light and CO$_{2}$ for photosynthesis, but at the same time, they lose water to the atmosphere through transpiration. To replenish water loss, plants entertain an elaborate network of xylem conduits that connect the water uptake tissues in the roots with the evaporating tissues in the leaves. Similarly to porous media, water loss causes tension in the leaves, which drives root–leaf water transport along the pressure gradient between the leaves and the roots, supported by cohesion between water molecules. This is commonly described as the cohesion-tension theory (Dixon and Joly 1895; Zimmermann et al. 1993; Tyree and Zimmermann 2002; Shi et al. 2020).

Plant water transport through the xylem can only be maintained if the pressure drop between roots and leaves is greater than the hydrostatic pressure drop due to the change in the gravitational potential between roots and leaves. The drier the soil, the larger the tension in the soil itself, requiring even more tension (or lower water pressure) in the leaves to ensure water transport (Dixon and Joly 1895). These pressures in the xylem are commonly so low that the system is believed to operate in a meta-stable state, where any air seeding in a vessel can cause embolism propagation, resulting in embolisms, which would make a vessel dysfunctional for water transport (Sperry 1986; Canny 1998). Here we refer to air seeding as any process that introduces air into the xylem (Tyree and Zimmermann 2002), such as air entry through pit membranes or wounds. 

The presence of embolized conduits reduces the efficiency of water transport, expressed as a decrease in hydraulic conductivity. The reduced hydraulic conductivity due to embolism has to be compensated for by an increased pressure gradient, that is, lower water pressures in the xylem, in order to maintain an adequate water transport rate. The lower pressure can lead to more embolized conduits, resulting in the positive feedback loop of runaway embolism propagation (Tyree and Sperry 1988; Hölttä et al. 2009).

The vulnerability of a plant to embolism spreading and loss of hydraulic conductivity is the primary focus of current plant hydraulic research (McDowell et al. 2019). Manymethodological advances have been made to measure the ‘per cent loss of hydraulic conductivity’ (PLC) under water stress, but the methods to measure the exact hydraulic conductivity values in plants or plant segments have received relatively little scrutiny (see e.g. Cochard et al. 2013; Venturas et al. 2017). While there is currently a method to measure plant vulnerability *in vivo*, micro CT, it does not measure an absolute hydraulic conductivity but a theoretical one (Pratt et al. 2020). Therefore, we will not expand on its details in this paper.

In the most common method for measuring plant hydraulic conductivity, one end of the plant sample is attached to a tube with above-atmospheric liquid pressure (e.g. a water reservoir that is elevated), while the other is exposed to atmospheric pressure, where water flows into a reservoir placed on a balance. The change in the weight of the downstream reservoir over time is used to calculate the flow generated by the hydraulic head. In this paper, we will refer to this method as the ‘Sperry method’ (Sperry et al. 1988; Canny et al. 2007; Torres-Ruiz et al. 2012). A slight modification to this method was employed by Tyree and Yang (1992), who added a balance under the supplying reservoir, allowing to quantify gains in sample water content during the refilling of desiccated samples under pressure. A similar technique to the Sperry method uses a high-pressure flow meter (HPFM), where pressure is generated on one side using compressed air to push water through a plant sample connected by flexible tubing. The flow is calculated from the pressure drop across a capillary located between the plant sample and the pressure generator. In this paper, we will refer to this method as the ‘HPFM setup’ (Tyree et al. 1993). The HPFM setup is mainly used for measuring the hydraulic conductivity of roots (Tyree et al. 1995; Tsuda and Tyree 2000). Yet another modification to the Sperry method was proposed by Kolb et al. (1996), who measured the hydraulic conductance of entire branches or root systems by placing them in a vacuum chamber and connecting the protruding stem with a water reservoir placed on a balance. In this setup, water is transported through the system by suction, and hydraulic conductance is calculated from the linear relationship between flow rate and vacuum pressure in the chamber. We will refer to this method as the ‘Kolb setup’.

A method that in theory enables conductivity measurements at negative liquid pressure is the Cavitron method (Alder et al. 1997; Cochard et al. 2013), where a plant sample with rotor cups on each end is placed in a centrifuge, and the pressure inside the twig is determined by the rotation rate of the centrifuge, whereas the pressure gradient depends on the position of water menisci in the rotor cups. The water level of the downstream rotor cup is fixed at the position of its outflow hole, while the water level in the upstream rotor cup is located further off-centre, thus creating a gradient in water potential between the two ends of the plant sample. During the experiment, the water level of the upstream rotor cup slowly recedes as water is transported through the plant sample towards the downstream rotor cup. The flow rate could be calculated by taking the change in water level in the upstream rotor cup over time and multiplying it with the cross-sectional area of water in the rotor cup. However, due to the unknown twig volume in the rotor cup, an exact flow rate, and thus hydraulic conductivity, is not routinely determined; only the relative loss in conductivity is recorded as rotation speed is increased.

Unfortunately, none of the above methods reproduces the situation *in planta*:

- In the centrifuge method, the pressure along the twig is non-linear, with the lowest pressure in the middle (Cochard 2002), rather than at the sink, as would be the case in a real plant.
- In all of the above methods, the pressure difference along the sample is held constant (atmospheric at the outflow side of the twig and above-atmospheric at the inflow side for the Sperry and HPFM setups), while the flow rate adjusts according to the hydraulic conductance of the twig. This is in contrast to natural conditions in a plant, where the system functions at sub-atmospheric or even negative liquid pressure and the pressure drop along the flow path adjusts to the hydraulic conductivity and the flow rate, the latter of which is determined by the transpiration rate (Venturas et al. 2017). This means that a sudden decline in hydraulic conductivity would cause a sudden drop in pressure, which is not the case in the above methods, which all control the pressure gradient across plant samples such that in the event of embolism propagation, the flow rate decreases, not the pressure, as would be expected in an intact plant.
- Most methods involve above-atmospheric pressures, so water stress cannot be induced while measuring hydraulic conductivity.

Except for the setup by Tyree and Yang (1992), none of the above methods quantifies changes in water storage of the plant sample, so it is not clear if the flow measured at one end of the sample constitutes through-flow or if part of the observed flow is due to emptying or re-filling water storage in the plant sample (Torres-Ruiz et al. 2012).

To fill these methodological gaps, in the present study, we aim to design an experimental setup for measuring hydraulic conductivity closer to natural conditions, with the following goals:

1. Flow rate controlled, pressure drop as response;
2. Suction-induced flow;
3. Ability to simulate water stress in the plant segment;
4. Ability to measure changes in storage.

## Materials and Methods

Below, we present two different setups that were designed to achieve the above-mentioned goals. One is an intuitive, low-cost vertical setup resembling an artificial plant. The second is a more controlled horizontal setup, improving on certain shortcomings of the vertical setup.

### Artificial plant setup

**Figure 1:**
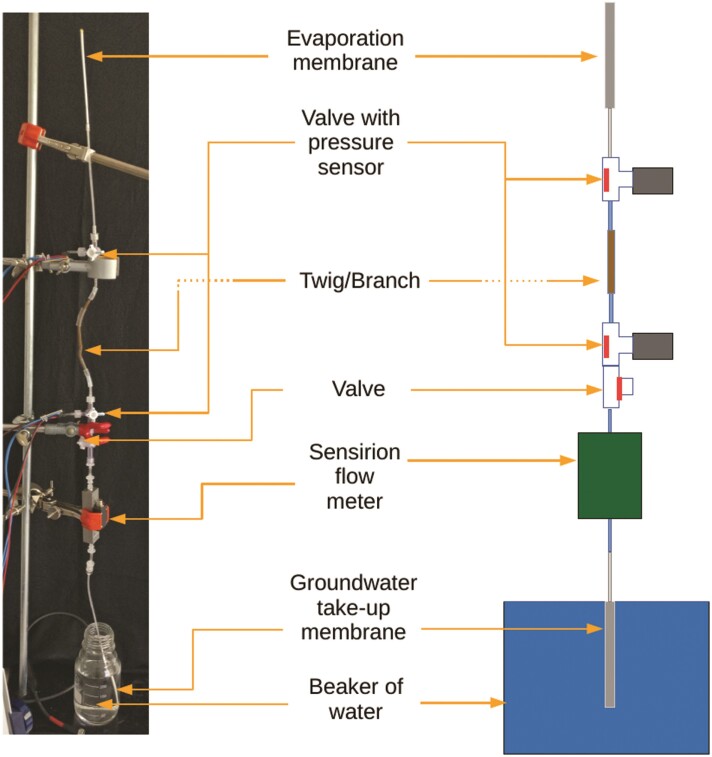
Setup for measuring hydraulic conductivity of twig samples using an evaporation membrane to drive flow.

The initial setup was designed to mimic a plant in the simplest form, containing one evaporating ‘leaf’, one ‘root’ immersed in a water reservoir, and connecting tubes where a plant segment is inserted in a vertical setup (Fig. 1). The root and leaf each are replicated by a Rhizon sampler (Rhizosphere Research Products B.V., Wageningen, The Netherlands), consisting of a membrane with pores of 5$\mu$m diameter. Pressure sensors (24PC; Honeywell, Morristown, NJ) are connected through t-valves on either side of the plant segment. A liquid flow meter (SLG-0150; Sensirion, Stäfa, Switzerland) is inserted in the flow path below the lower-pressure sensor. The water reservoir consists of a beaker filled with deionized (DI) water.

Evaporation from the leaf replica generates the suction necessary to pull water up all the way from the beaker, following the cohesion-tension principle. Note that the membranes need to be covered by a continuous water film, otherwise, air can enter through empty pores and water transpired at the surface is replaced by air, instead of water from below.

Initial filling of the system is performed without a twig in place, by placing both membranes in a beaker filled with DI water. A syringe is attached to one of the T-valves, at the position where the twig will be added. The syringe is pulled to fill the membrane and tubing with water. Turning the T-valve to only be open to the syringe and pressure sensor, the sensor is removed and water is pushed to fill that section and the sensor is re-attached. The process is repeated with the other side. Finally, the twig sample is attached and the setup is placed upright with one membrane in the beaker and the other held in the air using a stand where it begins to evaporate (Fig. 1).

### Horizontal syringe setup

**Figure 2:**
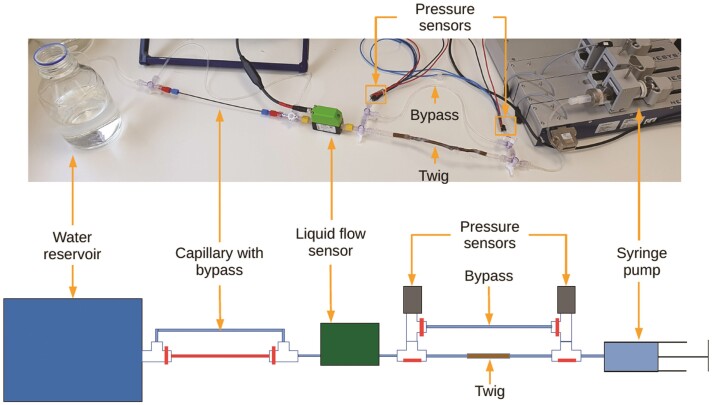
Horizontal syringe setup using a syringe pump (g) to control the water flow rate to and from the beaker (a). The twig (e) is inserted in the flow path to measure its hydraulic conductivity using flow (c) and pressure (d) meters. The bypass (f) is used to calibrate the sensors before an experiment. A capillary with bypass (b) is used as an example modification to lower the pressure.

The horizontal syringe setup (Fig. 2) consists of a syringe pump (neMESYS; Cetoni, Wiesenring, Germany) to control the water flow rate from a beaker passing through a twig sample. Pressure sensors on either side of the twig and a flow meter (SLI-0430; Sensirion, Stäfa, Switzerland) in the flow path allow for the measurement of hydraulic conductivity. Additionally, depending on the setting of the valves, flow can be passed through a capillary to increase flow resistance on the upstream side and hence reduce overall pressure in the system if desired. This setup will be referred to as the ‘syringe setup’.

The twig-less syringe setup is filled by pulling water, using the syringe, from the beaker to the syringe pump through the bypass connecting the pressure sensors, then the syringe is detached and the air within the syringe emptied before re-attaching it. The pressure sensors are individually removed to allow water to be gravitationally pushed to fill up to the connection point, where the sensors are then re-attached. 

### Calibration procedures

To avoid spurious results due to sensor drift, the following test and calibration procedures were carried out. The flow sensor is calibrated before each experiment as there can be instrument drift between experiments due to biological residue build-up, which can only be eliminated by periodic flushing with alcohol. For the calibration, water flow is redirected through the bypass around the sample with a direct path between the syringe and the water reservoir (Fig. 2). The known flow rates are generated using the syringe pump and correlated with measurements by the flow meter. The calibration is performed using a linear regression and applied to the data of the following experiment.

Pressure sensors were calibrated individually using the relation between pressure and volume obtained from the ideal gas law (Equation 1).


PV=nRT (1)

In Equation 1, *P* is the pressure (Pa), *V* is the volume of the gas (m${}^{3}$), *n* is the number of moles (mol), *R* is the gas constant (K mol Pa${}^{-1}$ m${}^{-3}$) and *T* is the temperature (K).

Assuming a completely sealed system (*n* is constant) and a constant temperature during the calibration, a change in the gas volume from ${V}_{\text{ref}}$ to ${V}_{\text{new}}$ would cause an inversely proportional change in pressure from ${P}_{\text{ref}}$ to ${P}_{\text{new}}$, such that:


$$P_{\text{new}}=\frac{P_{\text{ ref}}V_{\text{ref}}}{V_{\text{new}}}$$ (2)

The pressure sensors were attached to the syringe with a small tube and t-valve which was closed to the atmosphere; the internal volume of which was considered as the reference gas volume (*V*${}_{\text{ref}}$) and thus needed to be measured. To do so, we weighed the empty tube and t-valve, before and after filling it with water. By taking the weight difference between them and dividing it by the density of water, we obtained the reference gas volume.

The pressure sensor measures pressure relative to the atmosphere using two openings, one open to the atmosphere and one attached to the volume of interest. A membrane between the two openings bends with the difference in pressures and delivers a voltage based on the amount of inflection. With no difference (both at atmospheric pressure), no voltage is delivered and 0 V was measured. After filling the system with air at atmospheric pressure (assumed to be 101.3 kPa) and recording the measured sensor voltage (0 V), a known change in volume was applied using the syringe and the corresponding pressure was determined from Equation 2. This pressure, along with the measured sensor voltage, was recorded. This was repeated in several steps while increasing volume and decreasing volume again, in order to prevent any transient conditions (e.g. temperature or instrumental drift or air leaks) would influence the slope of the calibration. Both sensors were calibrated one after another on the same day, taking 15 min for each sensor. In order to determine if the values of the sensors drifted over time, a second calibration was done 2 years later. The data of both calibration sets were pooled together, resulting in a data series of 22 points for the bottom sensor, with pressures ranging between 2.56 and 101.3 kPa, and 21 points for the top sensor, with pressures ranging between 2.68 and 101.3 kPa. Linear least square fits through the data gave Pearson’s correlation coefficients of $ r >0.998$ for each sensor. Due to the high correlation coefficients for the pooled data, suggesting minimal instrument drift, the slope and intercept of the pooled calibration data were applied to all experimental data presented here. Furthermore, any biases that would remain from this method cancel out when calculating the pressure difference between the sensors ($\Delta P $) for measuring conductivity. To further counteract potential instrumental drift, before each experiment, the difference in pressure between the two sensors is measured at two points to remove any offsets. The first point is measured before an experiment when the sensors are at atmospheric pressure. The second point is the lowest attainable liquid pressure reachable with the setup, that is, the water vapour pressure, which is measured once the experiment has concluded. The latter is measured by closing valves such that the syringe is only connected to both sensors via the bypass and flow cannot occur. The syringe is set to pull at 250 $\mu$L min${}^{-1}$, and as in-flow is stopped, pressure lowers to the vapour pressure where gas bubbles form and the pressure no longer decreases. The flow is continued for 20 min. The water vapour pressure measurement occurs after the experiment as the measuring process fills the setup with gas. From the two points, a linear regression of the difference between the pressure sensors is calculated and a correction is applied to remove potential offsets.

### Sample collection and connection

All twig samples used in this paper were collected from *Fagus sylvatica* in Belval, Luxembourg, following Wheeler et al. (2013) to avoid having any artificial embolism propagation in the sample. The branch is cut from the tree and left to sit in a bag for at least 30 min. The branch is then re-cut under water, cutting at least one mean vessel length from either side of the sample to make sure that any embolism due to the initial cut is removed. For samples in this paper, 10 cm is removed from each end, as Buchmüller (1986) found that 40 $\%$ of the dry wood samples of *Fagus sylvatica* had a maximum vessel length of under 8 cm, with an additional 30 $\%$ of samples having a maximum vessel length between 8 and 16 cm. Any branches along the cut segment are removed under water and sealed with parafilm and/or silicon gel to avoid air entry. Flexible tubing is attached to both ends of the submerged twig, then removed from the water and connected to either side of the setup. The diameter of the samples varied between 3.8 and 4.2 mm. We did not measure the diameter for all the samples, thus a value of 4.0 mm was used for the calculations (see below).

### Conductivity calculation

Flow through a porous medium such as the twig xylem can be described using Darcy’s Law (Equation 3):


$$Q_{V}=\frac{kA\rho }{\mu L}\Delta P$$ (3)

where *Q_m_* is the mass flow rate (kg s${}^{-1}$), *K* is the intrinsic permeability (m${}^{2}$), *A* is the cross-sectional area of the whole twig (m${}^{2}$), $\Delta P $*L* is the pressure drop along the flow path (MPa), $\mu$ is the dynamic viscosity (MPa $\cdot$ s) and is the length of the segment (m). The mass flow rate can be converted to avolumetric flow rate (*Q_V_* , m${}^{3}$ s${}^{-1}$) by multiplying the mass flow rate by the density of water:


$$Q_{V}=\frac{Q_{m}}{\rho }$$ (4)

In the literature, the efficiency of water transport through a twig sample is expressed in different ways, which have different relations to intrinsic permeability (*K*) and are expressed in different units. For example, some authors use specific conductivity, which is affected by viscosity, *K_S_*$=\frac{ k \rho}{\mu}$ (kg m${}^{-1}$ MPa${}^{-1}$ s${}^{-1}$), others use specific conductance, which is affected by viscosity and sample length, *K_AS_*$=\frac{ k \rho}{\mu L }$ (kg m${}^{-2}$ MPa${}^{-1}$ s${}^{-1}$) (Caquet et al. 2009). In some cases, for example, Bär et al. (2018); Rosner et al. (2019), the units of specific conductivity were reported as ‘m${}^{2}$ MPa${}^{-1}$ s${}^{-1}$’, which are obtained by substituting the volumetric flow rate (Equation 4) for the mass flow rate in Equation 3.

Note that viscosity is temperature dependent, and therefore, specific hydraulic conductivity values should only be compared between measurements performed at similar temperatures. All the experiments presented in this paper were conducted in an air-conditioned lab around 21 ${}^{\circ}$C and humidity between 25 $\%$ and 40 $\%$. Continuous temperature measurements in a 500-mL beaker of water in the same lab revealed that the water temperature varied between 21 and 23 ${}^{\circ}$C over the duration of 2 weeks in September 2022, with a maximum temporal variation of 0.6 K in 2 h. Whenever temperature was needed for calculations, we used a temperature of 21 ${}^{\circ}$C.

For easier comparison with literature values, we do not report intrinsic permeability, but calculated the specific hydraulic conductivity of twig samples using the formulation of Sperry et al. (1988):


$$K=\frac{Q_{V}\rho }{\Delta P}\frac{L}{A}$$ (5)

where *K* is the hydraulic conductivity (kg m${}^{-1}$ MPa${}^{-1}$ s${}^{-1}$) and $\rho$ is the density of water (kg m${}^{-3}$). The pressure drop ($\Delta P $) was measured by pressure sensors on both sides of the twig, as described above. The length of the twig is measured as the distance between the centres of the cuts on each side. The cross-sectional area (*A*) is calculated from the stem diameter assuming a circular shape. The flow rate in the syringe setup is measured by the syringe pump (as a set flow rate out of the twig), and the flow sensor (as an instantaneous flow rate into the twig). Unless otherwise noted, the flow meter measurements were used to calculate conductivity in this paper as these resulted in more stable conductivity values **[see Supporting Information—Fig. S3]**.

### Experiments

In the first experiment, the artificial plant setup was left to evaporate to test the measurement of flow, pressure difference and conductivity change over time. Then, we attempted to produce runaway embolism propagation by adding a gas bubble to initiate embolism. The gas bubble was the width of the tubing and 1.5 times the width in length, and was added through a valve below the lower pressure sensor and rose to the twig while flow, pressure and hydraulic conductivity were being measured.

The remaining experiments were conducted using the horizontal syringe pump setup. First, the setup was compared with the current standard, the Sperry method, to verify if similar values of hydraulic conductivity are obtained using either method. For direct comparison, both methods were applied consecutively using the same sample. The Sperry method was applied by disconnecting the syringe pump (in Fig. 2), and letting the water drain freely while elevating the beaker at the other end of the setup (Fig. 2) to create the desired pressure difference. The beaker was elevated to 35.5 cm for 30 min, then to 73.5 cm for 30 minutes, then to 104.9 cm for 30 min and then returned to 73.5 cm for another 30 min, before returning to 35.5 cm for 30 min.

After this set of measurements, the beaker was placed back on the table, and the syringe pump was re-attached. Water was pulled through the sample (in the same flow direction as before) at flow a rate of 10 $\mu$L min${}^{-1}$ for 30 min, then at 20 $\mu$L min${}^{-1}$ for 30 min, then at 30 $\mu$L min${}^{-1}$ for 30 min, then again at 20 $\mu$L min${}^{-1}$ for 30 min, before returning to 10 $\mu$L min${}^{-1}$ for another 30 min.

The next experiment was designed to simulate water stress in plants using the syringe setup. Two types of water stress were simulated: (a) reduced water supply (e.g. due to soil moisture drought) and (b) increased leaf water demand (e.g. in the mornings, or due to wind gusts or sun flecks). To simulate soil moisture drought, water flow between the beaker and the twig was deviated through a capillary by turning the connected valves (Fig. 2), resulting in increased flow resistance upstream of the twig and hence reduced pressure. Increased water demand was simulated by increasing the flow rate induced by the syringe pump, increasing the pressure gradient along the twig.

The final experiment was designed to quantify the change in twig water storage between a relaxed condition (zero flow, e.g. at night) and flow under tension (e.g. during the day). In this experiment, the syringe setup was started in the same way as in the soil moisture drought experiment, that is, water was passed through a capillary before reaching the twig. When the measured flow rate into the twig became roughly steady, the syringe pump was stopped and the subsequent slow decay in flow rate was monitored until flow was no longer detected. The differences between the syringe pump flow and the flow meter signal were interpreted as the rate of change in twig storage.

## Results

### Conductivity measurement with artificial plant setup

Time in the graphs begins at 0 with the start of the experiment, which represents the first time flow was induced. This was either when the membrane was removed from water, the beaker was moved to a higher elevation, or the flow was started with the syringe pump.

**Figure 3:**
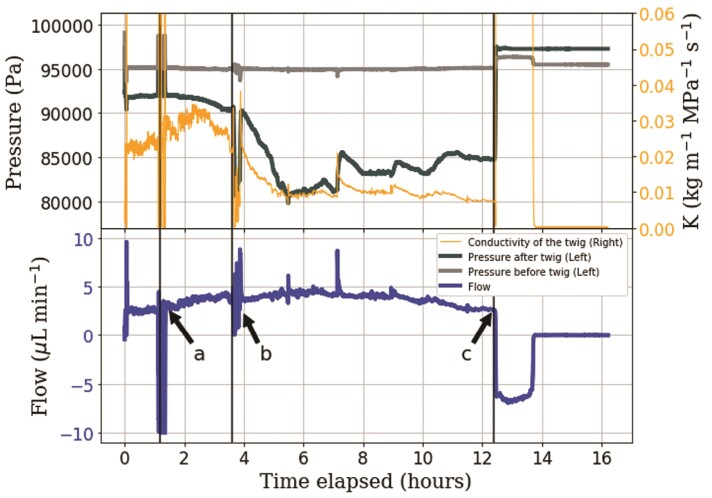
Flow, pressure and conductivity measurements of a 2.2-cm *Fagus sylvatica* sample in the artificial plant setup. Air bubble was added below the lower pressure sensor ‘a’, and reached the sample at time ‘b’. Air entered the membrane at the top and flow reversed at ‘c’.

A 2.2-cm-long twig was attached to the artificial plant setup and left to evaporate. The flow rate started at 2.5 $\mu$L min${}^{-1}$ and increased steadily to 4.0 $\mu$L min${}^{-1}$ over the first 3.7 h of the experiment. During this time, the pressure above the twig slowly decreased from 93 to 91.5 kPa (Fig. 3). The conductivity of the sample increased from 0.023 to0.025 kg m${}^{-1}$ MPa${}^{-1}$ s${}^{-1}$ over the first hour. The air bubble was added at 1.5 h (Fig. 3a) while conductivity continued to increase to a peak of 0.033 kg m${}^{-1}$ MPa${}^{-1}$ s${}^{-1}$ at the 2.3-h point and then decreased again to 0.027 kg m${}^{-1}$ MPa${}^{-1}$ s${}^{-1}$ at 3.7 h, when the bubble reached the sample. At this point, flow dropped to 0 and pressure decreased rapidly to 80.7 kPa (Fig. 3b), at which point the air bubble began passing through the twig. After 15 min, we were able to observe air bubbles coming out of the twig on the upper side, indicating that at least part of the gas in the introduced bubble passed through the whole twig and left it again at the other end. The pressure returned to the previous 91.5 kPa, and flow resumed at 4 $\mu$L min${}^{-1}$. The flow remained relatively stable for the next 1.5 h while both pressure and conductivity decreased markedly (from 91.5 to 80.7 kPa and from 0.021 to 0.01 kg m${}^{-1}$ MPa${}^{-1}$ s${}^{-1}$, respectively) during this time. Both pressure and conductivity stayed relatively steady for the next 6 h until air entered the upper membrane from outside at 12.5 h (Fig. 3c). The upper pressure increased and flow reversed, draining water from the setup above the twig, until the water meniscus stopped at the top of the twig.

### Horizontal syringe setup versus Sperry method

**Figure 4:**
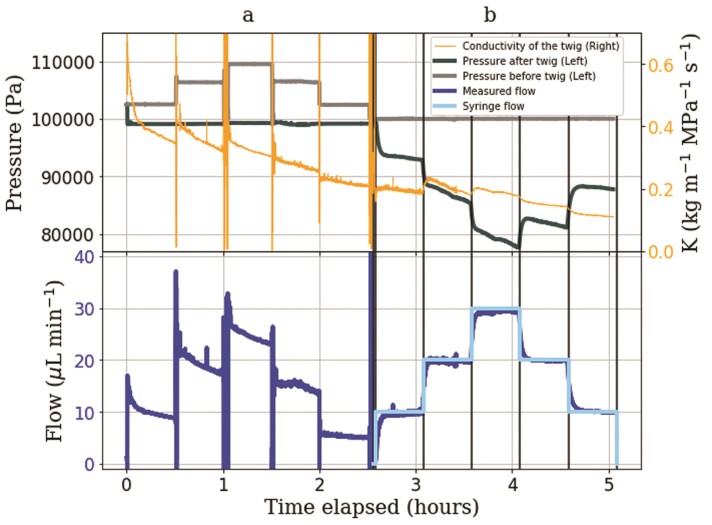
Pressure, flow and conductivity measurements of a 10.2-cm twig sample over time. Section ‘a’ represents measurements when water is pushed through a twig sample using controlled pressure differences (Sperry method). Section ‘b’ represents measurements when water is pulled through the same sample in the same direction using a syringe pump (syringe setup).

During the Sperry method (Fig. 4a), conductivity decreased steadily by 66 % over the total 2.5 h. When the pressure gradient was increased at 0.5 h, there was a step increase in conductivity from 0.36 to 0.41 kg m${}^{-1}$ MPa${}^{-1}$ s${}^{-1}$. Similar increases in conductivity were observed every time the pressure gradient was increased, but only one of the two-step decreases in pressure gradient resulted in an evident decline in conductivity (at 2 hrs, not at 1.5 hrs). Also, when the method switched from the Sperry method to the syringe method at 2.5 h, the hydraulic conductivity was not affected and remained at 0.21 kg m${}^{-1}$ MPa${}^{-1}$ s${}^{-1}$ during the transition. Over the 2.5 h of the syringe pull (Fig. 4b), conductivity also decreased (decreased 50 % over 2.5 h), but more slowly than during the Sperry method. Whenever the flow rate was increased, there was a step-wise increase in conductivity, and whenever the flow rate was decreased, there was a slight step-wise decrease in conductivity.

When the different hydraulic heads were applied in the Sperry method (Fig. 4a), the pressure was constant and the flow rate decreased over time. However, when different flow rates were applied in the syringe method (Fig. 4b), it was the pressure that decreased over time while the flow was constant.

### Simulating water stress

**Figure 5:**
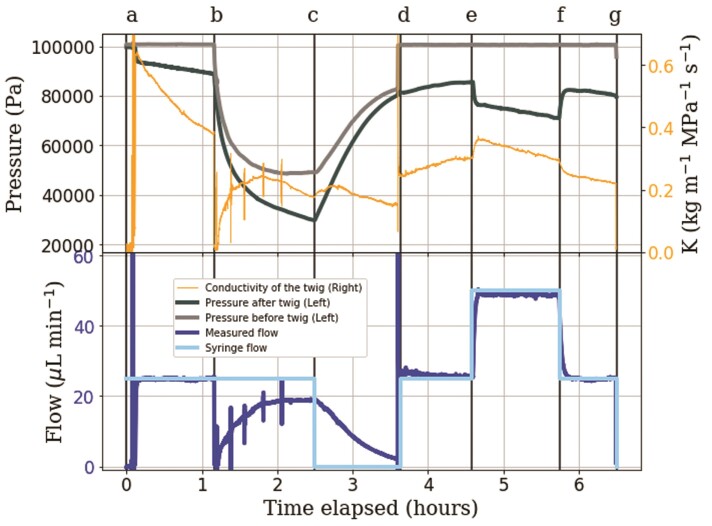
Time series of pressure, flow and conductivity of a 13.5-cm twig when simulating different sorts of water stress. At ‘a’, a constant pull through the twig at 25 $\mu$L min${}^{-1}$ is applied and flow goes around the capillary. At ‘b’, the flow is led through a capillary upstream of the twig. At ‘c’, the syringe pump is stopped. At ‘d’, conditions are similar to ‘a’. At ‘e’, flow is further increased to 50 $\mu$L min${}^{-1}$. At ‘f’, the system returned to the same conditions as ‘a’ and ‘d’. The experiment ends at ‘g’.

In Fig. 5, the letters ‘a’ through ‘g’ indicate a change to the experimental setting. In Segment ‘a–b’, a constant pull was applied through the twig at 25 $\mu$L min${}^{-1}$ with no flow through the capillary, representing a steady evaporation of the leaf during the day at the unrestricted water supply. This reference scenario was established in Segments ‘a–b’, ‘d–e’ and ‘f–g’, for direct comparison to the stress conditions.

Drought stress is simulated at ‘b’, where the flow was redirected through a capillary upstream of the twig. In Segment ‘b–c’ , flow initially stopped and pressure before the twig decreased sharply, followed by a decrease in pressure after the twig, and slow recovery of flow and conductivity, which reached 0.23 kg m${}^{-1}$ MPa${}^{-1}$ s${}^{-1}$ before declining again. When the syringe pump was stopped at ‘c’, flow decreased from 19 to 3 $\mu$L min${}^{-1}$ over the course of an hour, in Segment ‘c–d’, while pressures increased. Conductivity remained similar to that in Segment ‘a–b’, around 0.20 kg m${}^{-1}$ MPa${}^{-1}$ s${}^{-1}$.

At ‘d’, the syringe pump was turned on again and the capillary bypassed, as in Segment ‘a–b’. When the pump was turned on, the flow rate increased instantly, accompanied by a step increase in conductivity from 0.15 to 0.26 kg m${}^{-1}$ MPa${}^{-1}$ s${}^{-1}$. Note that the measured flow rate was initially even higher than the syringe pump flow rate in Segment ‘d’.

An increase in water demand was simulated at ‘e’, where water flow through the twig was increased from 25 to 50 $\mu$L min${}^{-1}$. The flow change was immediately reflected by the flow meter and pressure after the twig decreased suddenly from 85 to 74 kPa, while the conductivity increased from 0.30 to 0.35 kg m${}^{-1}$ MPa${}^{-1}$ s${}^{-1}$, followed by a steady decline in Segment ‘e–f’ from 0.35 to 0.30 kg m${}^{-1}$ MPa${}^{-1}$ s${}^{-1}$.

**Figure 6:**
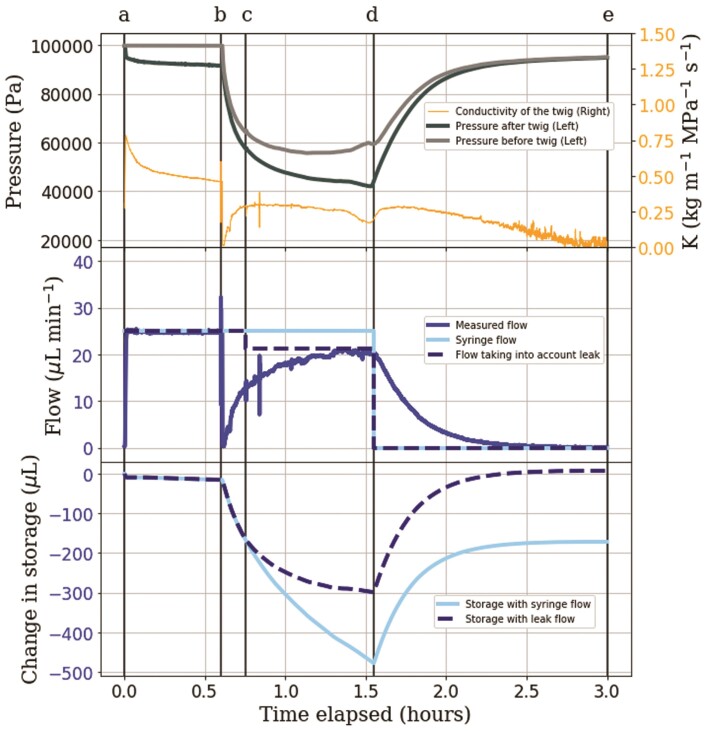
Graph of pressure, flow, conductivity and storage changes when simulating water stress on a 11.4-cm twig. At ‘a’, a constant pull through the twig at 25 $\mu$L min${}^{-1}$ was applied and flow went around the capillary. At ‘b’, flow was lead through the capillary. At ‘d’, flow was stopped. The experiment ended at ‘e’. Change in storage was calculated as the cumulative difference between measured flow and syringe flow. An air leak was assumed to have formed at ‘c’, see the main text.

Once the original flow of 25 $\mu$L min${}^{-1}$ was re-established at ‘f’, pressure after the twig increased again to almost itsoriginal value at ‘e’, whereas conductivity showed another step decrease from 0.29 to 0.26 kg m${}^{-1}$ MPa${}^{-1}$ s${}^{-1}$. Conductivity in Segment ‘f–g’ decreased steadily from 0.26 to 0.22 kg m${}^{-1}$ MPa${}^{-1}$ s${}^{-1}$. The overall decrease in conductivity throughout the experiment could be seen in Fig. 4-6.

### Twig water storage

To better understand if the deviations between syringe pump and flow meter flow rates observed in the previous experiment were related to changes in twig water storage, Segments ‘a–d’ were repeated with a new twig, but with a longer period without syringe pump flow at the end. Changes in storage were calculated as the cumulative difference between the flow rates at the syringe pump and the flow meter.

At the start of the storage experiment (Fig. 6 Segment ‘a–b’), the same pattern of declining conductivity as in the stress experiment was found. When the flow was passed through the capillary at ‘b’, the measured flow rate briefly declined to 0, followed by a steady recovery, reaching a steady rate of 21.7 $\mu$L min${}^{-1}$ at ‘d’, 3.3 $\mu$L min${}^{-1}$ lower than the syringe flow. Interpreting the difference in flow rates as a change in twig water storage would suggest that the amount of water in the twig decreased by 475 $\mu$L at ‘d’. When the syringe pump was turned off (Segment ‘d–e’), the flow meter kept recording flow into the twig, suggesting re-filling of the twig water storage until the flow ceased entirely after another 1.5 h. At this point, our calculation would suggest a remaining twig water deficit of 175 $\mu$L (light blue line in the bottom panel of Fig. 6).

The difference between the steady-state flow rate measured by the flow meter at ‘d’, and the flow rate imposed by the syringe pump suggests that a leak occurred in the system, as a steady-state difference cannot be explained by changing storage. In addition, we observed a bubble in the syringe, confirming the presence of an air leak. The leak was assumed to have formed at ‘c’ due to a slight kink in the pressure, which would likely occur when air entry is initiated. In the absence of more detailed information, the air entry rate was assumed constant in Segment ‘c–d’ and to be equal to the flow rate difference between the steady-state flow at ‘d’ and the syringe flow. Using the flow rate based on the assumed air entry rate, storage would have decreased by a maximum of 300 $\mu$L at ‘d’ and the end storage at ‘e’ would have been 7 $\mu$L larger than the initial twig water storage at ‘a’.

## Discussion

In this paper, we presented two different methods for measuring hydraulic conductivity under suction and flow-controlled conditions. The first method is an intuitive representation of an artificial plant as an evaporating leaf, transport tissues and a root, but it does not offer precise control of the flow. In numerous experiments using this method (not all shown here), we found that stable flow could be maintained for many hours, but invariably, sudden, catastrophic failure occurred by air entry through the evaporating microporous membrane after many hours. An interesting feature of this setup is its educational value, as its vertical orientation and components are intuitively associated with key macroscopic components of the plant hydraulic system. In this evaporation-driven experimental setup, the flow rate continued even when the flow was temporarily constricted, providing the inspiration for a fully flow-controlled horizontal setup using a syringe pump to control the flow (second method).

This second method is of more scientific value, as it allows for more precise control of flow rate and quantification of twig storage changes as flow rates are known for both sides of the twig. Estimations of twig water storage changes can also be enabled by adding a second balance in the Sperry setup (Tyree and Yang 1992), but here we avoid evaporation from the beakers, which could cause artefacts in the flow measurements. Our method combines the advantage of quantifying changes in twig water storage with the advantage of measuring conductivity by suction instead of above-atmospheric pressure, as proposed by Kolb et al. (1996), who pointed out that the large pressures needed to measure samples with low conductance (e.g. due to partial embolism) would lead to re-filling of embolized vessels and therefore over-estimate conductance. Therefore, measurements under suction are expected to lead to more accurate hydraulic conductivity values. Since the pressure is measured on both sides of the twig in our setup, we can add an additional resistance (such as a capillary) to the upstream side of the twig to reduce the pressure in the twig. Note that although a sub-atmospheric pressure could also be achieved by elevating the twig relative to the water reservoirs in the setup by Tyree and Yang (1992), for the pressure drop of 70 KPa shown in Fig. 5, the twig would have to be elevated to a height of 7 m, so that setup can only be used for very mild reductions in pressure. In essence, our second method combines the advantages of both setups presented by Tyree and Yang (1992) and Kolb et al. (1996), with the additional bonus of controlling flow while the pressures adjust, which is more representative of a situation where flow is driven by evaporation.

The two setups presented here are able to provide different scientific insights and highlight different challenges for the quantitative understanding of flow through plant segments. In the artificial plant setup, where we introduced an air bubble below the twig, we were able to see a sudden decline in liquid pressure on the leaf side as the bubble reached the twig, but then, surprisingly, as the pressure difference reached a threshold of 15 kPa (Fig. 3b), the bubble started entering the twig and pressure returned to its original value within tens of minutes. Contrary to expectations that air entry would lead to reduced or discontinued flow through the hydraulic system, flow and evaporation continued at the original rate throughout this experiment, with only short-term perturbations when the bubble was introduced and when it passed through the twig. This was despite a marked decrease in hydraulic conductivity from 0.035 to 0.010 kg m${}^{-1}$ MPa${}^{-1}$ s${}^{-1}$ during the experiment. It is not clear if the decline in hydraulic conductivity was caused by the added air or independent of it, as the decreasing trend was observed before the bubble reached the twig, and the same trend continued after it had passed through the twig. Note that the behaviour documented here was only observed with a twig that was shorter than it saverage xylem vessel length (here a twig of 2.2 cm was used, cf. an average xylem vessel length of under 8 cm for *Fagus sylvatica* (Buchmüller 1986)). When a longer twig of 12.3 cm was used **[see Supporting Information—Fig. S2]**, flow declined to zero shortly after the bubble reached the lower twig end after 4.2 h. In this case, the bubble did not pass through the twig, blocking the flow completely and eventually leading to air entry through the evaporating membrane and failure after 5.9 hours **[see Supporting Information—Fig. S2]**. This illustrates that there was no vessel longer than 12.3 cm present in the sample, and that air could not pass from one vessel to another, such that flow was completely blocked. In the artificial plant setup, any gas bubbles transported with the water accumulated at the top of the artificial leaf, presumably allowing water supply to the evaporating sites only through a thin film along the membrane. This increased resistance to flow likely created a large pressure drop between the bulk water and the evaporating sites at the tip, eventually resulting in air entry at the top of the membrane. When the air-entry value of the evaporating membranes was tested without a twig and air bubbles, we found sudden air entry at liquid pressures between 35 and 57 kPa, which is consistent with the reported pore sizes of the membrane **[see Supporting Information—Fig. S1]**. The accumulation of air bubbles at the evaporating sites and subsequent hydraulic failure highlights the importance of mechanisms to not let air bubbles accumulate in the hydraulic system, even in the absence of negative liquid pressures.

To avoid the hydraulic failure of the system due to the accumulation of air in the evaporation membrane and fluctuations in flow rate due to variations in lab humidity or air movement around the evaporating membrane, we developed the horizontal syringe setup, which also improves the previous design by arranging the setup horizontally, and hence avoiding the offset between pressure sensors due to gravitational potential and ensuring that the pressure difference measured by the sensors does actually represent the pressure drop along the twig. Note that branches can grow horizontally, so a horizontal setup is not less ‘natural’ than a vertical one. The replacement of the evaporation membrane by a syringe pump eliminates the pressure limitation due to the membrane’s air entry pressure, and fluctuations in flow caused by fluctuations in evaporation from the membrane. For the same reason, the use of a rhizon on the water uptake side was also abandoned. The original idea was to place the rhizon in a porous material to simulate the reduced liquid pressure exerted by the soil, but with the rhizons used, the pressure could not be lowered below the air entry pressure of the membrane. Instead, a capillary was used to reduce the pressure on the receiving side of the twig. The syringe pump allows the flow rate to be precisely controlled through either suction or pushing, as opposed to evaporation from the membrane. The horizontal syringe setup and the Sperry method gave comparable values for the hydraulic conductivity of the same sample (Fig. 4). Furthermore, the overall trend in the conductivity over time is maintained when swapping between the two methods. The step increase and decrease patterns, when changing pressure difference or flow rate, were seen in both methods, suggesting that the results are likely not of methodological nature, but biological. The specific hydraulic conductivity was measured between 0.20 and 0.60 kg m${}^{-1}$ MPa${}^{-1}$ s${}^{-1}$, 3–4.5 times lower than literature values: 2.5 kg m${}^{-1}$ MPa${}^{-1}$ s${}^{-1}$ in Rosner et al. (2019) using the Sperry method, and1.83 kg m${}^{-1}$ MPa${}^{-1}$ s${}^{-1}$ in the Xylem functional traits database (https://xylemfunctionaltraits.org/) Choat et al. (2012). As pointed out by Kolb et al. (1996), the higher conductivity values reported in the literature could be due to the filling of empty vessels when samples were flushed with water at high pressures.

In all our experiments, we observed continuous declines in hydraulic conductivity during measurements, which have also been reported before and largely attributed to microbial growth (Sperry et al. 1988). To avoid the decline in hydraulic conductivity and to mimic xylem sap, it has been suggested to add HCl, KCl or oxalic acid to the perfusing solution (Sperry et al. 1988; Kolb et al. 1996; Nardini et al. 2011). However, a decline in K under large pressure gradients even with de-gassed KCl solution and bacteriocide was found in a study by Bonetti et al. (2021). This decline could easily be investigated further using the syringe setup, especially the question of whether the decay is due to pressure or flow rate, which cannot be done with any of the current setups.

Here we also note that the previously reported declines were over timescales of tens of hours (Sperry et al. 1988), whereas our experiments only lasted a few hours. Therefore, we used DI water and were surprised to see the largest declines within the first half-hour in our horizontal experiments (Figs. 4–6). Note that in our vertical experiments, where the flow rate was much lower, the initial decline in conductivity was not observed (Fig. 3 and **Supporting Information—Fig. S1**). This could imply that the decline in hydraulic conductivity was not so much due to microbial growth as to the accumulation of bubbles at the pit membranes (Canny et al. 2007). More experiments using different flow rates would be needed to separate these processes more clearly.

The comparable hydraulic conductivity measurements between the Sperry method and the horizontal syringe setup, along with approximately similar results to literature values confirm that the horizontal Syringe method is a valid alternative to the Sperry method for measuring hydraulic conductivity. In addition, the syringe setup was able to measure hydraulic conductivity while simulating water stress conditions. Simulated soil moisture stress caused a decrease in the pressure on both sides of the twig sample (Fig. 5), which is not possible using the Sperry or HPFM methods, as they rely on fixed pressure gradients and above-atmospheric pressure. Surprisingly, our experiment showed that an increase in flow rate increased the conductivity of the sample, both at constant pressure difference (Sperry method) and constant flow rate (syringe method). Conversely, step decreases in flow rate resulted in step reductions in conductivity in three out of four cases in Fig. 4. Since flow rates are positively correlated with pressure in the Sperry method (increase in pressure drives flow), but negatively correlated with pressure in the syringe method (increase in suction drives flow), the combination of both methods allows the conclusion that the sample’s conductivity indeed depends on the flow rate, not the liquid pressure. The positive correlation between flow rate and hydraulic conductivity was also found in Fig. 5, at the transitions marked as d, e and f. More targeted experiments on different species could shed light on potential mechanistic reasons for this behaviour.

Another advantage of the syringe pump setup is that the flow rate is measured on both sides of the twig, giving additional information about the state of experiments. When pulling water through a sample, the water leaving the twig is determined by the syringe pump, while the flow entering the twig is measured by the liquid flow meter at the other end. In our experiments, it has enabled the detection of leaks or changes in the twig’s storage (Fig. 6). The following processes can cause a deviation between the measured flow (*Q_m_*) and the syringe pump flow rate (*Q_s_*):

1. Changes in water storage of the system between the flow meter and the syringe pump. This could result in *Q_m_*$>$*Q_s_* or *Q_m_*$<$*Q_s_*.
2. Evaporation of water from the twig. This would result in *Q_m_*$>$*Q_s_*.
3. Air seeding or exsolution of gas between the flow meter and the syringe pump. This would result in *Q_m_*$<$*Q_s_*. Air bubbles should become visible in the tubes or the syringe pump in this case.

To quantify the storage changes in the tubing, we ran an experiment similar to Figure 6, but without a twig. The results suggested that a significant deviation in flow rates between the syringe pump and flow meter could only be maintained for a few minutes and the total change in storage was less than 60 $\mu$L **[see Supporting Information—Fig. S4]**. Since we never observed persistently greater flow meter values compared to the syringe pump, we can exclude a significant contribution of evaporation from the twig. The only occurrences of *Q_m_*$>$*Q_s_* were found for a limited time after reductions in flow rate, implicating changes in storage as the reason. In two cases, we documented persistently *Q_m_*$<$*Q_s_*, both under conditions of low pressure, and associated with the accumulation of gas in the syringe, indicating that air might have entered. In general, if *Q_m_* deviates from *Q_s_*but then converges, this indicates that the system storage is adjusting to a new steady state. In Figs. 5 and 6, we found clear indications of changes in storage, some of which were followed by indications of temporary leaks or gas exsolution periods (persistently *Q_m_*$<$*Q_s_* in Fig. 5b–c, and Fig. 6c–d). In Fig. 6, we calculated the change in storage from the cumulative sum of *Q_s_*$-$*Q_m_* and used the steady value of *Q_s_*$-$*Q_m_* in Fig. 6c to quantify the hypothesized air entry rate. Remarkably, when accounting for this air entry, assumed to occur only at a liquid pressure below 55 kPa (based on a slight bump in the pressure and flow data at this threshold), the storage deficit gradually returned to zero 1.5 h after switching the syringe pump off. This indicates the capacity of the twig to reversibly reduce and replenish its storage depending on the flow rate and pressure applied.

This elastic storage component may also be the reason for the so-called ‘passive water uptake’ commonly found when using the Sperry method, which is then subtracted from the measured flow rates to achieve more consistent results (Torres-Ruiz et al. 2012). Since the magnitude of the ‘passive water uptake’ increases with the xylem tension prior to the experiment (Table 1 in Torres-Ruiz et al. 2012), it is likely that the underlying mechanism is the same as that causing water flow into the twig in our experiments at zero syringe pump flow rate after the water stress treatments (Figs. 5 and 6). The dynamic decay of this spontaneous water uptake observed in our experiments is consistent with the interpretation that it is likely related to a relaxation of elastic tissues (Zweifel et al. 2001). However, since the dynamics of flow during a conductivity measurement is usually not reported, we cannot tell how far the ‘passive water uptake’ analysed by Torres-Ruiz et al. (2012) is indeed related to the elastic relaxation seen in our experiments, and if it was, how a constant rate of ‘passive water uptake’ could be deduced from such a dynamically decaying curve. Fortunately, the ability to measure flow rate on both sides of the twig in our setup gives us a clear indication of any artefacts in the flow measurements, and to our surprise, conductivity calculations based on the measured flow rate of water into the twig and the pressure gradient along the twig produced consistent conductivity values even during moderate emptying or re-filling of the twig water reservoir (see e.g. conductivity values before and after Point d in Fig. 6). Note that the change in storage of our system without a twig is an order of magnitude smaller than the change in storage observed in the presence of a twig **[see Supporting Information—Figs. S4 and S5]**, so we can rule out an artefact due to elasticity in our system.

The experiments presented here were not designed to gain any particular scientific insights, but to illustrate the capabilities and potential limitations of the newly presented methods. The main limitation of both methods is that the liquid pressure cannot be lowered sufficiently to induce a significant loss of conductivity during a flow measurement. Even if the valve upstream of the twig is closed while the syringe pump is sucking, liquid pressure only decreases down to the saturation vapour pressure of the water in the tubes, that is, around 3 kPa at 25 ${}^{\circ}$C, at which point cavitation occurs, triggered by any gas bubble in the system, including those inside the pressure sensors. Conducting flow and pressure measurements below this pressure, or even at negative pressures in the MPa range, as expected in plants, would require the removal of all gas bubbles and any cavitation nuclei in the system, which has so far only been achieved in microscopic systems (Wheeler and Stroock 2008; Pagay et al. 2014). Nevertheless, even at the modest range of sub-atmospheric pressures attainable in the current setup, we have been able to observe transient changes in twig water storage lasting for up to an hour, suggesting that this setup could be used to not only measure the hydraulic conductivity of plant segments very accurately, but also gain a better understanding of the role of water storage in the plant hydraulic system.

## Conclusions

Current methods of measuring the hydraulic conductivity of plant segments are based on controlling a pressure gradient and pushing water through samples, which does not reflect natural water transport processes in plants, that is, suction-driven flow with a pressure gradient determined by the flow rate imposed by leaf water demand. Here we describe two new experimental approaches to measure hydraulic conductivity using suction and a controlled flow rate. The artificial plant setup, consisting of an artificial root, an artificial leaf and a plant segment in the flow path between the two, is well suited for educational purposes, as its components are intuitively comparable to real plant organs. The syringe pump setup, where the evaporating artificial leaf is replaced by a syringe pump, is more versatile for conducting scientific experiments. Our detailed tests of the setup confirmed that the conductivity values obtained are similar to those measured with the traditional Sperry method when similar flow rates are used. However, due to the use of a flow meter before the twig and syringe pump controlled suction at the other end, the setup enables quantifying changes in twig water storage. We found that simulating water stress by increasing flow resistance at the source or flow rate at the sink both resulted in the transient withdrawal of water from the twig, which was largely reversible, that is, the twig replenished its storage to the original value when the original flow conditions were restored.

This enables unique insights into the interplay between pressure, flow rate, hydraulic conductivity and water storage in plant segments.

## Supporting Information

The following additional information is available in the online version of this article –

**Figure S1.1**. Altered vertical experimental setup to determine minimum attainable pressure before air entry into membrane.

**Figure S1.2**. Flow and pressure measurements of the artificial plant setup.

**Figure S2.** Flow, pressure, and conductivity measurements of 12.3 cm fagus sylvatica sample in the artificial plant setup.

**Figure S3**. Time series of hydraulic conductivity calculated using the syringe flow and the measured flow for a 13.5 cm fagus sylvatica twig while simulating different sorts of water stress.

**Figure S4**. Flow and change in water storage of the horizontal syringe method when stressing the system.

**Figure S5**. Change in water storage of the system when creating stress condition with and without twig sample.

## Sources of Funding

This study was supported by the Luxembourg National Research Fund (FNR) - ATTRACT programme (A16/SR/11254288).

## Contribution by Author

L.K. constructed the experimental setups, and carried out the experiments and data analysis. Conceptualization of the experimental design, interpretation of the results and writing of the text was done jointly between L.K. and S.J.S.

## Acknowledgements

We would like to acknowledge Michael Roderick, Steven Jansen, and Roman Zweifel for providing inspiration and discussion about current methods and their relevance in plant hydraulics research. We also thank Frank Minette, Oliver O’Nagy, and François Barnich for their creative technical support.

## Conflict of Interest Statement

None declared.

## Data Availability

All data and analysis code is available at https://doi.org/10.5281/zenodo.7322605.

## Supplementary Material

plad024_suppl_Supplementary_Material

## Notes

**Form and Function. Chief Editor:** Kate McCulloh
