# Supplementary material for: A new experimental setup to measure hydraulic conductivity of plant segments
Source: AoB Plants. 2023 Jun 15;15(4):plad024. doi: 10.1093/aobpla/plad024 (PMC10418303; doi:10.1093/aobpla/plad024)
Supplement: plad024_suppl_Supplementary_Material [file plad024_suppl_supplementary_material.pdf]

SI

March 20, 2023

1 Membrane experiment for minimum pressure

The following section refers to the vertical experimental setup shown in Fig. 1 of the main text.

An experiment was conducted without a twig, just to determine the minimum sustainable liquid pressure in the system before air enters through the membrane. After the valve was closed, essentially blocking water supply while evaporation from the upper membrane was ongoing (Fig. S1.1), the pressure kept decreasing until it reached a minimum, followed by a temporary increase in pressure before sudden air entry occurred through the top membrane. Over three trials, air entered at pressures between 49.5 kPa and 56.9 kPa. When air entered through the top membrane, the flow direction reversed until all the water drained into the lower part of the setup.

The limit due to this air entry of the setup was tested in the second experiment. If the water pressure falls below a certain point, air enters through the membrane's pores and stop water from flowing to the top. The pressure difference required between the air and the liquid for air to enter is referred to here as the "air entry value".

The expected air entry value for the membranes can calculated based on the pressure drop across the water-air interface in the largest pore, using a form of the Young-Laplace equation (Young et al., 1807):

$\Delta P_i = \frac{2\sigma_w}{r_p}$

where σ_w is the surface tension of water (N m^{-1}), r_p is the radius of the pores in the membrane (m), and P_{ae} is the air entry value (Pa). Taking a surface tension of water at 20°C of 0.07286 N m^{-1} and the pore radius of 2.5×10^{-6} m reported for the membranes, the expected pressure drop would be 58288 Pa, and thus, the theoretical lowest pressure the water in the membrane can reach before air entry happens at atmospheric pressure would be $101325 - 58288 = 43037$ Pa. Note that liquid pressures below this values would be expected to allow air to enter, but as long as the air entry rate is slower than the evaporation rate, liquid pressures could still decrease further below these values. This is consistent with our experimental results where a minimum pressure of 30 kPa was achieved, followed by a progressive increase in pressure and then catastrophic failure at a pressure of 35 kPa (Fig. S1.2). It is likely that air entry first occurs slowly in the form of bubbles, which accumulate at the top of the system, leading to the afore-mentioned additional pressure drop along the membrane and eventually air entry through all pores in the top part of the membrane.

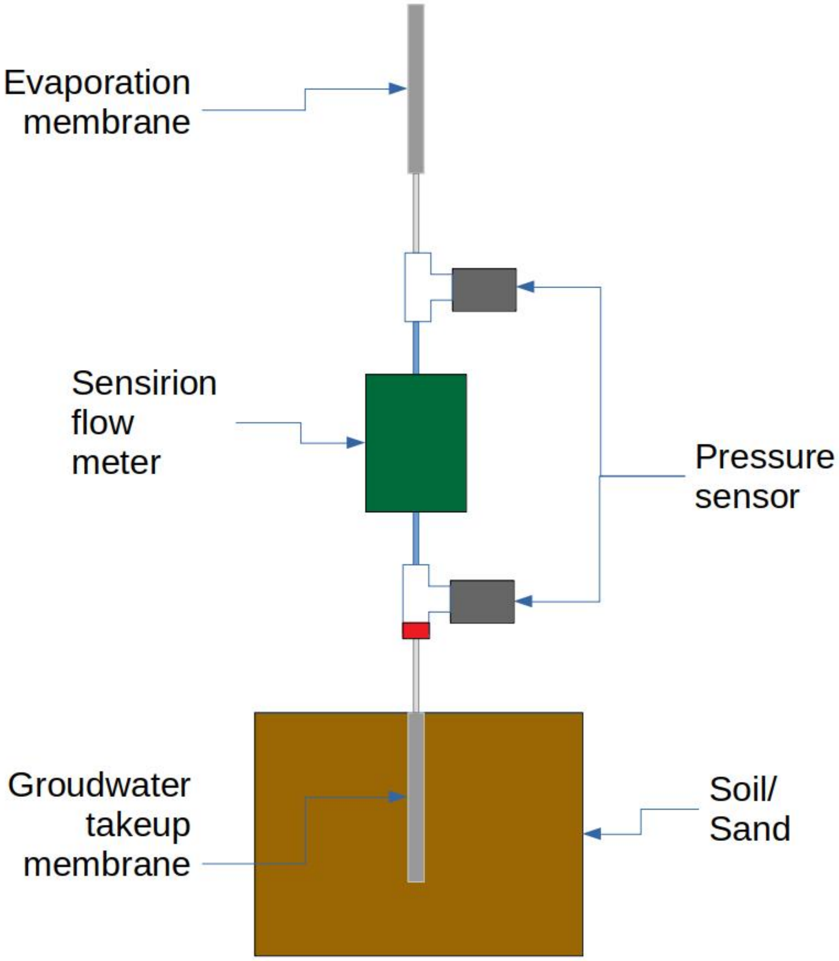

Figure S1.1 - Altered vertical experimental setup to determine minimum attainable pressure before air entry into membrane.

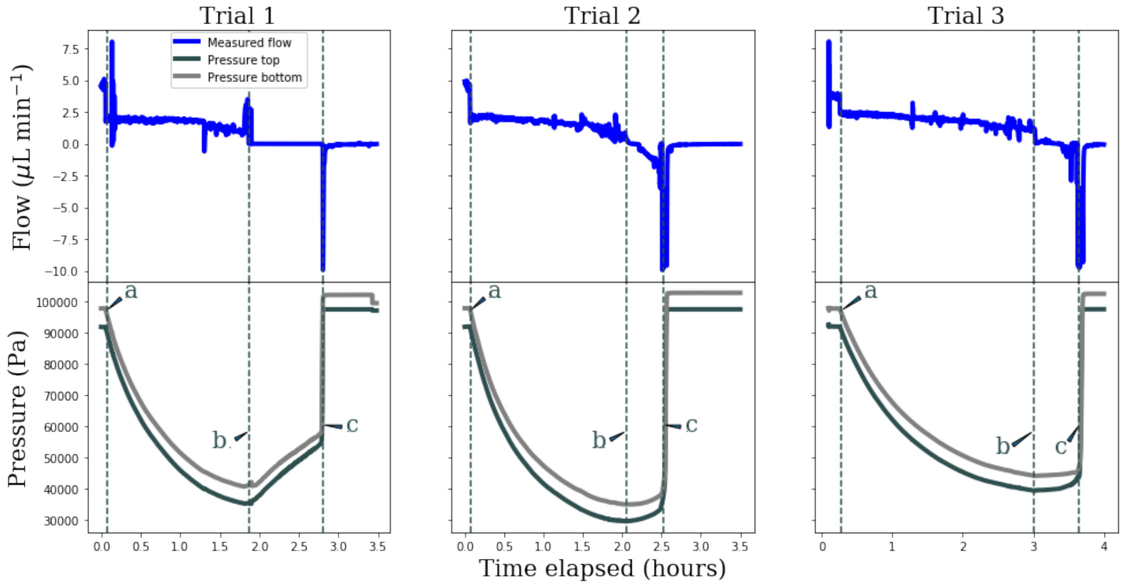

Figure S1.2 - Flow and pressure measurements of the artificial plant setup. In the three trials, the valve below the pressure sensors was closed at 'a', leading to a decrease in pressure to a minimum value of 'b'. The membrane continued evaporating until air entered the membrane's pores and flow reversed at 'c'.

2 Membrane experiment with long twig

The measurements of conductivity, flow, and pressure of a 12.3cm twig undergoing the same bubble experiment as in Fig. 3 of the main text.

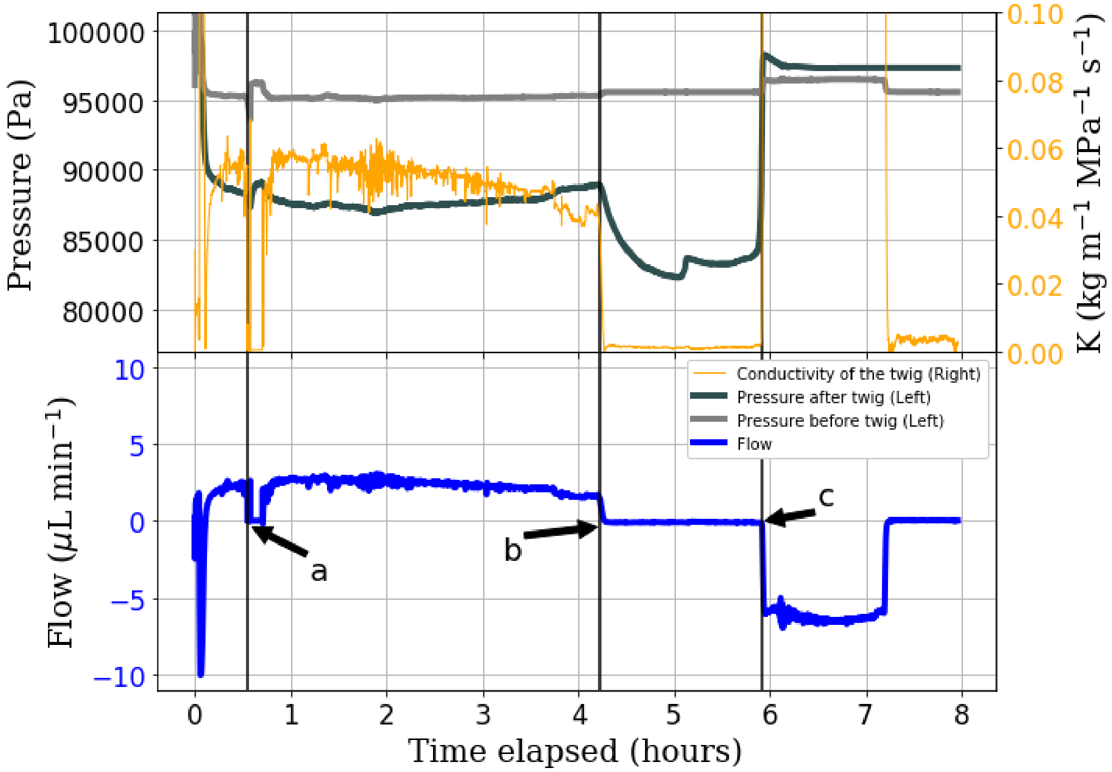

Figure S2 - Flow, pressure, and conductivity measurements of 12.3 cm fagus sylvatica sample in the artificial plant setup. Air bubble was added below the lower pressure sensor 'a', and reached the sample at time 'b' where flow stopped and pressure decreased until air entered the membrane at the top and flow reversed at 'c'.

3 Conductivity using syringe vs measured flow

The conductivity measurements using both syringe and flowmeter values for the experiment of Fig. 5 in the main text.

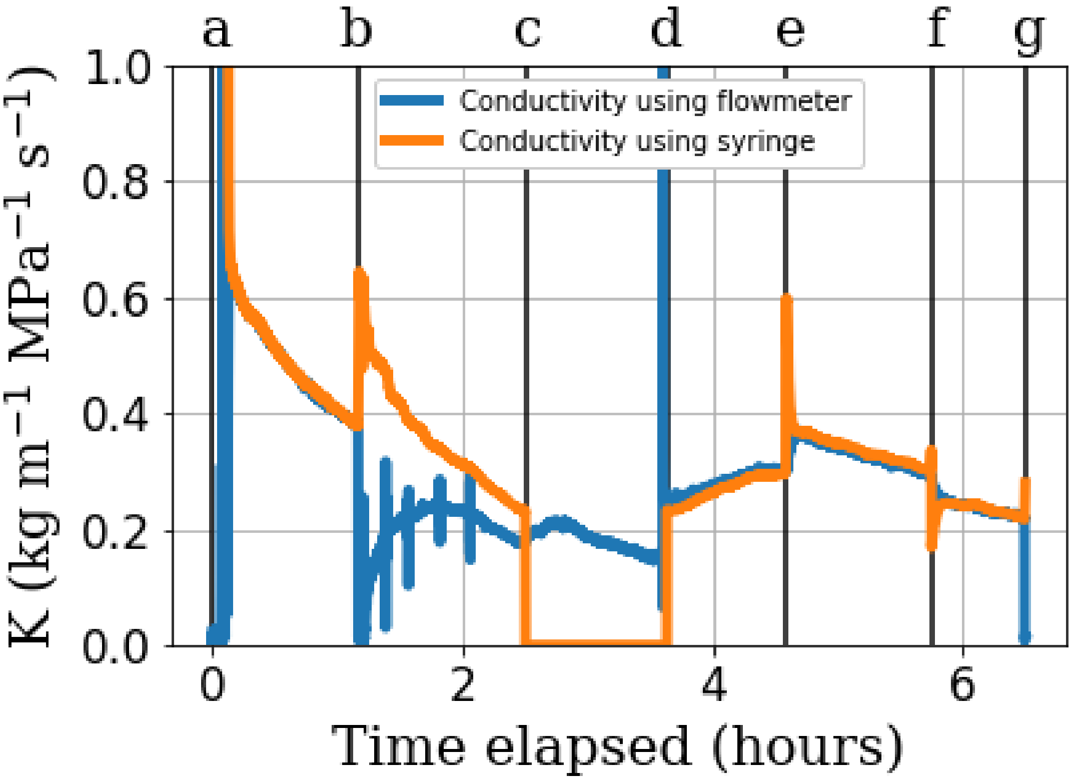

Figure S3 - Time series of hydraulic conductivity calculated using the syringe flow and the measured flow for a 13.5 cm fagus sylvatica twig while simulating different sorts of water stress. At 'a', a constant pull through the twig at 25 μL min^{-1} is applied and flow goes around the capillary. At 'b', flow is lead through a capillary upstream of the twig. At 'c', the syringe pump is stopped. At 'd', conditions are similar to 'a'. At 'e', flow is further increased to 50 μL min^{-1} and returned to 25 μL min^{-1} at 'f'. The experiment ends at 'g'.

4 Change in water storage of the system

Here we look at the change in water storage of the experimental setup without a twig and only the capillary. The capacitance of the system is used as a correction for twig storage in Fig. 6 of the main text.

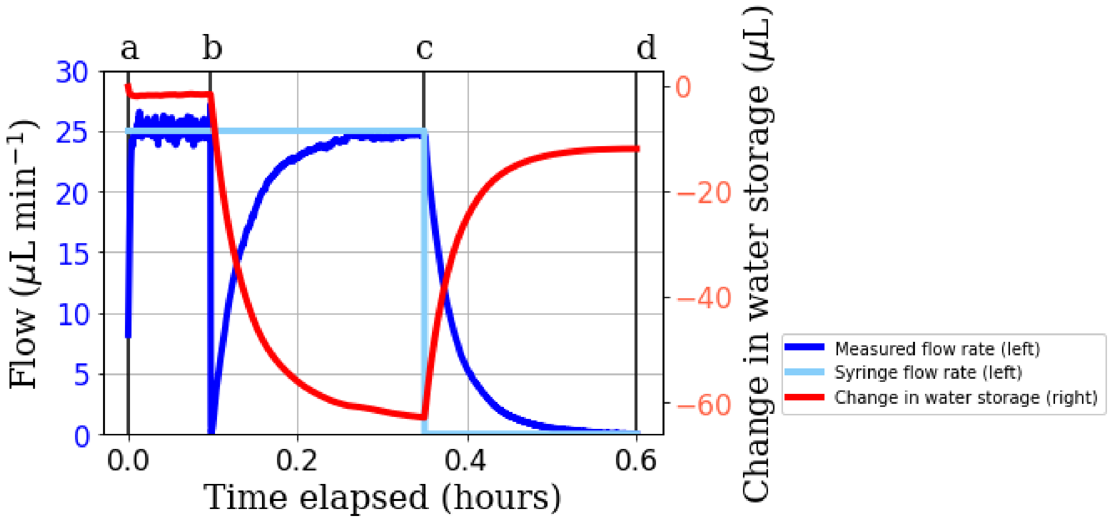

Figure S4 - Flow and change in water storage of the horizontal syringe method when stressing the system. At 'a', a constant pull through the twig at 25 μL min^{-1} is applied and flow goes around the capillary. At 'b', flow is lead through a capillary. At 'c', the syringe pump is stopped.

5 Change in water storage of system vs twig experiment

Here we show comparison of the system's change in water storage to that of the twig from Fig. 6 in the main text, presenting the magnitude difference between the storage values.

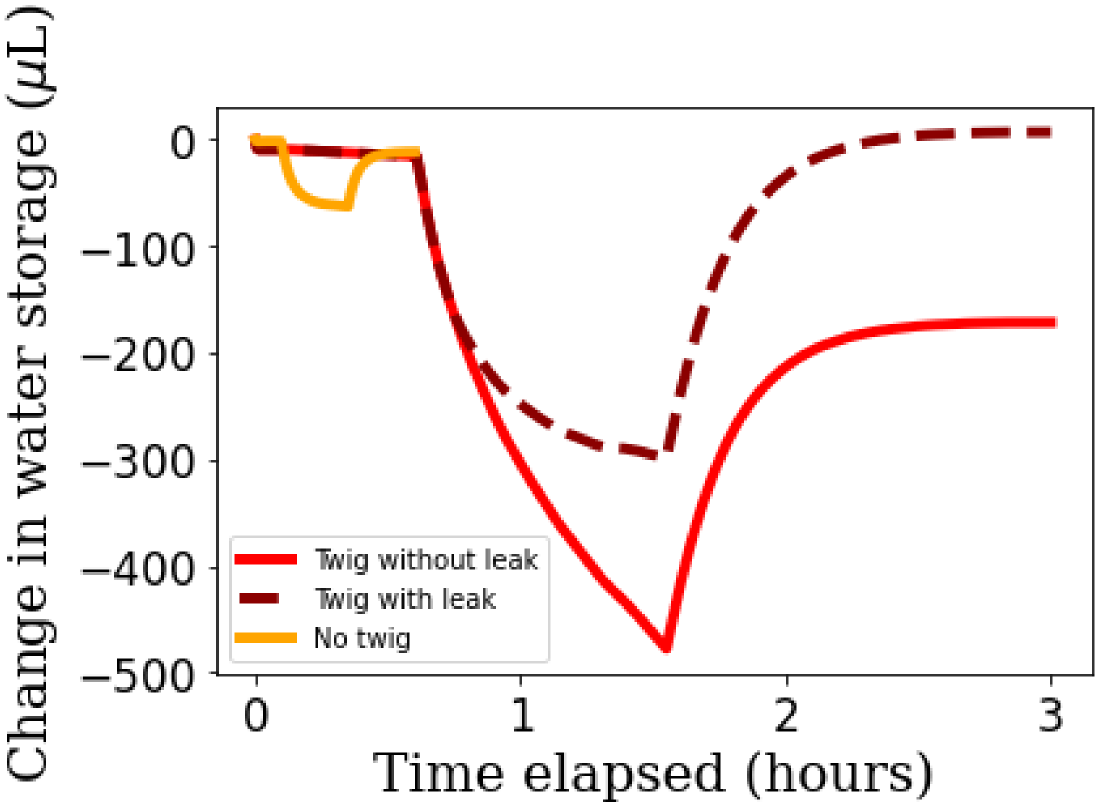

Figure S5 - Change in water storage of the system when creating stress condition with and without twig sample. The changes in water storage of the twig are represented with and without taking the leak into consideration in the calculations.
